# Supplementary material for: Structural and Transport Properties of Thin InAs Layers Grown on InxAl1−xAs Metamorphic Buffers
Source: Nanomaterials (Basel). 2025 Jan 23;15(3):173. doi: 10.3390/nano15030173 (PMC11819693; doi:10.3390/nano15030173)
Supplement: Supplementary file 1 [file nanomaterials-15-00173-s001.zip › nanomaterials-3418587-supplementary.pdf]

Supplementary Material:
Structural and transport properties of thin InAs layers grown on $In_xAl_{1-x}As$ metamorphic buffers

Giulio Senesi [1], Katarzyna Skibinska [1], Alessandro Paghi [1], Gaurav Shukla [1], Francesco Giazotto [1], Fabio Beltram [1], Stefan Heun [1] and Lucia Sorba [1,*]

[1] Istituto Nanoscienze-CNR and Scuola Normale Superiore, Piazza San Silvestro 12, 56127 Pisa, Italy; g.senesi5@studenti.unipi.it (G1.S.); katarzyna.skibinska@nano.cnr.it (K.S.); alessandro.paghi@nano.cnr.it (A.P.); gaurav.shukla@nano.cnr.it (G2.S.); francesco.giazotto@nano.cnr.it (F.G.); fabio.beltram@sns.it (F.B.); stefan.heun@nano.cnr.it (S.H:); lucia.sorba@nano.cnr.it (L.S.)

* Correspondence: lucia.sorba@nano.cnr.it

1. Asymmetry evaluation in different azimuthal directions

To rule out the presence of tilt in Sample K and in the InAs on metamorphic buffer samples, we performed measurements in different azimuthal directions to test the symmetry in the scans in [110] and [1-10] directions.

Sample K has been measured using the symmetric (004) reflection planes, with azimuthal angles (Φ) ranging from 0° to 270°. All four scans are shown in Figure S1. To address the presence of any asymmetry in the peak shifts, we calculated the lattice constants as if no tilt was present. The perpendicular lattice constants are reported in Table S1 for different Φ values. As summarized in Table S1, all measured lattice parameters are comparable within the experimental error for both the InAs peak and the shoulder peak. This suggests that no asymmetry and thus no tilt is found in sample K for different sample orientations. We also performed grazing incidence asymmetric (115) scans with Φ equal to 0°, 90°, 180°, and 270°. However, the InAs and shoulder peak intensities were less than 15 and 10 cps, respectively. Since the noise was around 7 cps, the signal-to-noise ratio does not allow to establish a peak shift. We notice also that, for (004) scans, even if the InAs layers have a nominal thickness of 100 nm, the intensity of the InAs peak of sample K is a factor 2 and 5 lower with respect to sample E and J, respectively. This low signal intensity of sample K is consistent with our explanation of an InGaAs layer formation, resulting in a thinner InAs film.

As for sample K, the symmetric (004) scans were measured in the 0° and 90° azimuthal directions also for sample E, to rule out any tilt in the InAs on metamorphic buffer samples. The InAs peak shift in the 90° direction is less than 10 arcsec different from the 0° direction, resulting in the same perpendicular lattice constant for the two directions within less than 0.001 Å, even less than the estimated error (0.002 Å). This suggests that no asymmetry is present in the 0° and 90° directions of (004) scans. Furthermore, since the InAs signal of sample E is larger than that of sample K, also (115) glancing incidence asymmetric scans were performed for 0°, 90°, 180°, and 270° azimuthal angles (see Figure S2). As reported in Table S2, no significant variations were found for the different azimuthal directions. All calculated lattice parameters (and peak shifts) are comparable within the experimental error.

We conclude that no azimuthal asymmetry was found in any of the grown samples (A-K). Therefore, we do not expect any tilt angle to be present.

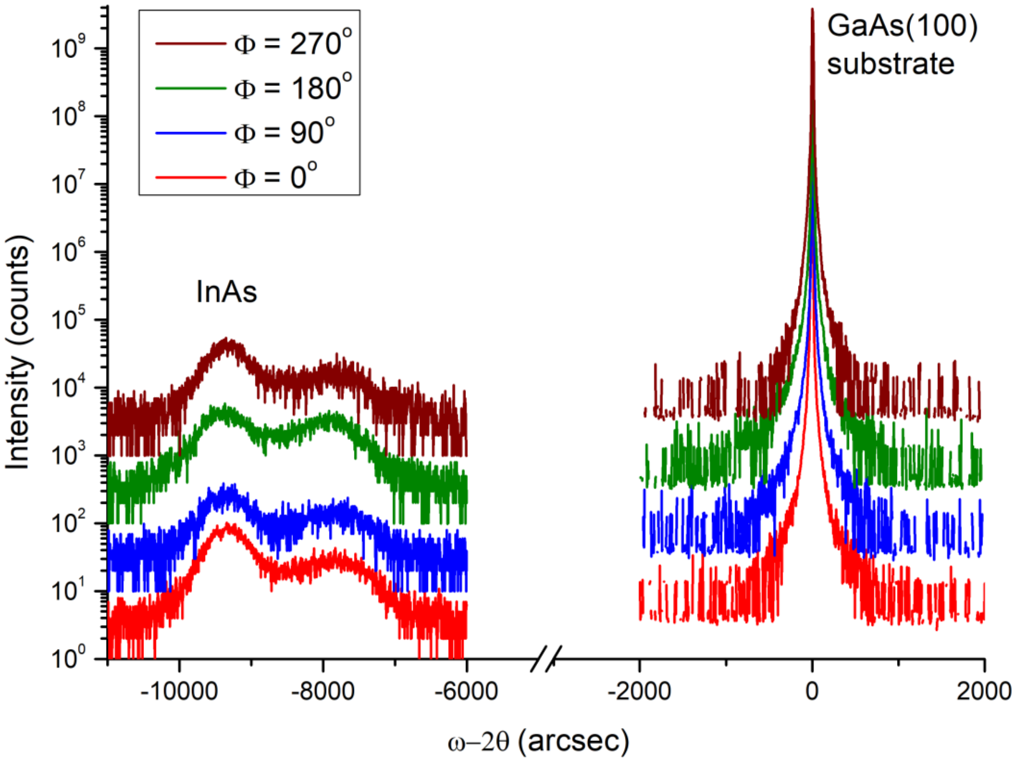

Figure S1. Symmetric (004) ω-2θ scans of sample K obtained along different azimuthal angles (Φ) ranging from 0° to 270°. An integration time of 1 sec was used for the InAs and shoulder peaks while for the rest of the scan range, this time was 0.1 sec.

Table S1. Summary of the measured perpendicular lattice constants of the InAs and the shoulder peak for symmetric (004) scans at different azimuthal angles (Φ) for sample K. All measured lattice constants are comparable within the experimental error, and no asymmetry is found.

**Φ [°]**	**$a_{\perp}$ InAs peak [Å]**	**$a_{\perp}$ shoulder peak [Å]**
0	6.082 ± 0.003	6.012 ± 0.010
90	6.085 ± 0.003	6.015 ± 0.008
180	6.083 ± 0.003	6.010 ± 0.009
270	6.083 ± 0.003	6.011 ± 0.011

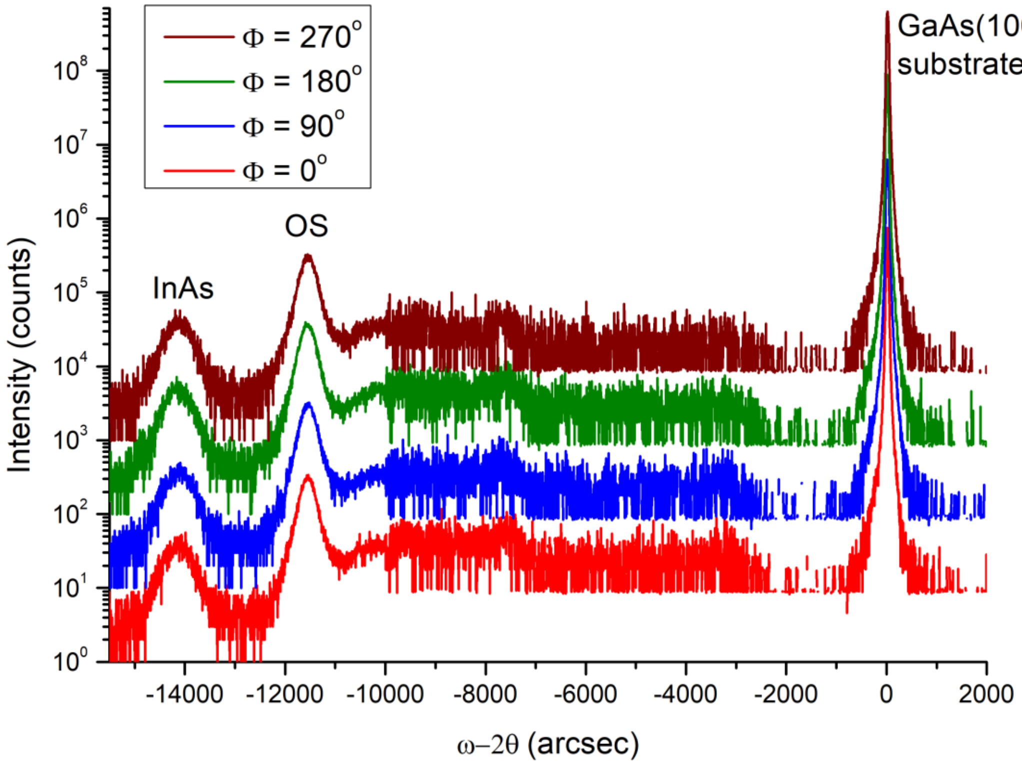

Figure S2. Asymmetric (115) ω-2θ scans of sample E obtained along different azimuthal angles (Φ) ranging from 0° to 270°, in glancing incidence configuration. An integration time of 1 sec was used for the InAs and overshoot peaks while for the rest of the scan range, this time was 0.1 sec.

Table S2. Summary of the measured perpendicular and parallel lattice constants of the InAs and the overshoot (OS) peak for asymmetric glancing incidence (115) scans at different azimuthal angles (Φ) for sample E. All the measured lattice constants are comparable within the experimental error, and no asymmetry is found.

**Φ [°]**	**$a_\perp$ InAs peak [Å]**	**$a_\perp$ OS peak [Å]**	**$a_{//}$ InAs peak [Å]**	**$a_{//}$ OS peak [Å]**
0	6.0834 ± 0.0044	5.9976 ± 0.0022	6.0834 ± 0.0044	5.9976 ± 0.0022
90	6.0834 ± 0.0044	5.9976 ± 0.0023	6.0834 ± 0.0044	5.9975 ± 0.0023
180	6.0831 ± 0.0044	5.9975 ± 0.0022	6.0831 ± 0.0044	5.9975 ± 0.0022
270	6.0833 ± 0.0044	5.9976 ± 0.0022	6.0833 ± 0.0044	5.9976 ± 0.0022

2. Optical Microscopy Images

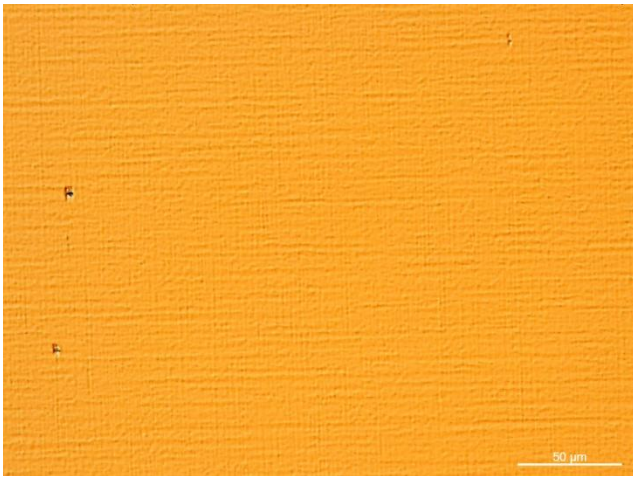

(a) InAs/InAlAs (V/III = 7, T_g = 500°C)

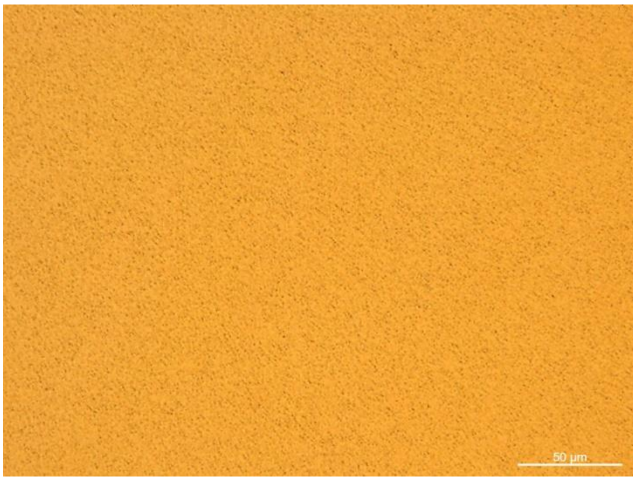

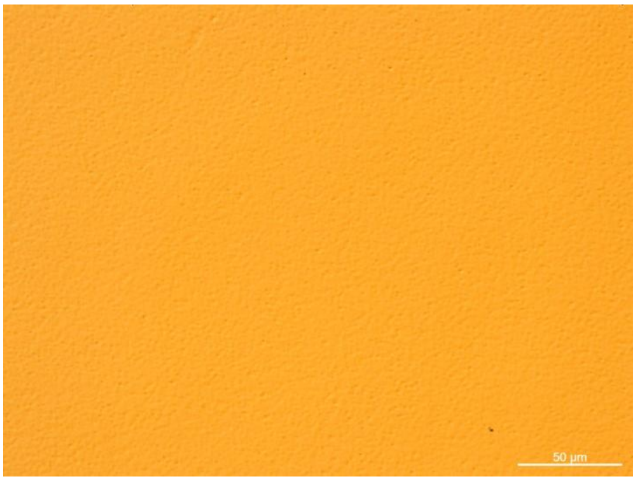

(b) InAs/GaAs(100) (V/III = 7, T_g = 500°C) **(c)** InAs/GaAs(100) (V/III = 10, T_g = 560°C)

Figure S3. Nomarski optical microscopy images. **(a)** Optical microscopy image of the surface of sample E grown with a V/III flux ratio equal to seven and an InAs growth temperature of 500°C. The average surface defect density of sample E is 11 ± 4 mm^{-2}. For all InAs epilayers grown on metamorphic buffers, the average surface defect density is 10 ± 2 mm^{-2}. We notice that the surface of sample E and samples (A-I) show a crosshatch pattern. This is a common feature of lattice-mismatched heteroepitaxy samples and was already reported for InAs/InGaAs Quantum Wells on GaAs(100) [1]. **(b)** Optical microscopy image of the surface of sample J grown with a V/III flux ratio equal to seven and an InAs growth temperature of 500°C. The average surface defect density of sample J is greater than 5 x 10^5 mm^{-2}. **(c)** Optical microscopy image of the surface of sample K grown with a V/III flux ratio equal to ten and an InAs growth temperature of 560°C. The average surface defect density of sample J is 9 ± 7 mm^{-2}.

References

1. Arif, O.; Canal, L.; Ferrari, E.; Ferrari, C.; Lazzarini, L.; Nasi, L.; Paghi, A.; Heun, S.; Sorba, L. Influence of an Overshoot Layer on the Morphological, Structural, Strain, and Transport Properties of InAs Quantum Wells. *Nanomaterials* **2024**, *14*, 592, https://doi.org/10.3390/nano14070592.
